# Attitudes and behavior related to performance-enhancing substance use among elite Saudi football players

**DOI:** 10.1186/s13102-019-0149-1

**Published:** 2019-12-05

**Authors:** Mohammed Al Ghobain

**Affiliations:** 1 Department of Medicine, Ministry of National Guard-Health Affairs, Riyadh, Saudi Arabia; 2 King Abdullah International Medical Research Center, Riyadh, Saudi Arabia; 3 King Saud bin Abdulaziz University for Health Sciences, Riyadh, Saudi Arabia

## Abstract

**Objective:**

To investigate the attitudes, beliefs and behavior related to performance enhancing substances (PES) use in elite Saudi football players.

**Method:**

A cross-sectional survey was conducted. Using a systematic random sample of elite Saudi male football players, the standard World Anti-doping Agency (WADA) Social Science Research Package questionnaire was distributed to 408 players.

**Results:**

The overall prevalence rate of PES use was 3.9%, with the overall prevalence rate of doping susceptibility 17.1%. PES use or doping susceptibility is strongly correlated but negatively associated with morality and cheating measures (*p* <  0.011, the estimate is − 0.139), threat or deterrence appraisal (*p* <  0.001, the estimate is − 0.301) and beliefs about the reference group’s endorsement (*p* < 0.001, the estimate is − 0.213) but not with legitimacy perceptions (*p* = 0.513) and beliefs about the benefits of doping (*p* = 0.678). The strongest relationship was found between threat or deterrence appraisal (*p* < 0.001), and beliefs about the reference group’s endorsement of PES use (*p* < 0.001).

**Conclusion:**

Morality and cheating measures, threat or deterrence appraisal and beliefs about the reference group’s endorsement are the main predictors for PES use in Saudi Arabia.

## Background

The use of performance-enhancing substances (PES) by athletes (doping) is a prohibited practice, but prevalent globally. Two of the following three criteria must be met for a substance to be considered as PES: (1) The substance increases or has the potential to increase performance; (2) the substance represents an actual or potential health risk to the athlete; and (3) the substance violates the spirit of sport.

Using a combination of questionnaires and models of biological parameters, the current prevalence of intentional doping in elite athletes is estimated as 14-39%. This variation in the range is related to different types of sport and athletes. It is estimated that approximately 1-2% per year is the result of doping control tests [1]. However, the prevalence in Saudi Arabia and other Arab countries is under-researched. Previously, we reported a prevalence rate of 4.3% in a countrywide survey with 1142 athletes from all regions of Saudi Arabia. The main reason reported for using prohibited substances was to improve performance (69%). Higher rates of using prohibited substances (doping) among Saudi athletes were associated with a lower education, being younger than 20 years, previous use of food supplements, and lack of punishment awareness [2].

To understand doping behavior and to develop preventive and educational programs for athletes, just estimating of doping prevalence is insufficient. It is essential to understand the psychological variables, as well as the beliefs and attitudes the athletes use as their motives to use or not use PES are diverse and complex. Behavioral factors influencing the use of PES vary and have been studied from different perspectives. For example, Strelan et al. concluded that the criminal decision-making theory and cost-benefit analysis are the main contributing factors for using PES [3]. Studying 116 elite Australian football and soccer players, Strelan et al. (2006) reported that the most predictive factor for athletes` PES use is moral beliefs and health concerns, not drug testing [4].

The World Anti-Doping Agency (WADA) reviewed publications investigating the attitudes and behavior related to PES use. They found that the vast majority of attitudinal research was descriptive and used cross-sectional designs, which is not appropriate for planning educational and preventive programs to prevent doping in sport [5]. As a result, WADA proposed the Social Science Research Package, a research package aimed to facilitate National Anti-Doping Committees and agencies in investigating their athletes’ beliefs, attitudes and behaviors of doping using a standard questionnaire. It is derived from the Sport Drug Control Model (SDCM) where the overall objective is to provide evidence of the influences on athletes’ doping related attitudes and behaviors to develop policy and practice in the real world. SDCM is an Australian model prepared by Donovan et al., providing a valuable tool to assess factors influencing PES use. PES use in this model is influenced by six major inputs: (1) threat appraisal (deterrence factor) (2) benefit appraisal, (3) reference group opinions, (4) personal morality, (5) perceived legitimacy of doping laws, and (6) personality factors [6].

The objective of this study is to investigate the attitudes, beliefs, behavior, and social factors in relation to PES use in a sample of elite Saudi football players using the WADA Social Science Research Package.

## Methods

### Study design

A cross-sectional countrywide survey using a systematic random sample of all elite Saudi male football players above the age of 16 years was conducted. An Elite Player is defined as a player who has a contract as a professional player at national or international level. The study was conducted by distributing the standard WADA Social Science Research Package questionnaire to 408 participants attending different sport clubs, stadiums, sports fields, and playgrounds affiliated with the Saudi Football Federation and the General Sport Authority throughout all regions of the country including Riyadh, Eastern, Northern border, Qassim, Hail, Jouf, Tabuk, Madinah, Makkah, Baha, Asir, Jazan and Najran.

Players were selected using a random sample selection technique that selected a proportion (*n* = 408) of the participants of the total number of registered elite football players (16,779). Recruiting players involved approaching them at events or after training and inviting them to participate in a face-to-face survey, (the interviewer provides the questionnaire to a player to self-complete). Written consent was obtained after explaining the aims and objectives of the study. Anonymity was ensured as the participant’s name was not recorded and the data were kept confidential to protect privacy. Data were not used for purposes other than the objectives of the study. The study protocol received ethical approval from the Ethical Research Committee (IRB) of King Abdullah International Medical Research Center, Riyadh, Saudi Arabia.

The questionnaire was translated to Arabic, reviewed, and validated by experts from the members of Saudi Anti-Doping Committee. It was pilot tested to ensure clarity and customized according to local needs. Some questions were found to be inappropriate or difficult to understand, and were omitted. The items contained in the questionnaire represented the following concepts based on the standard WADA Social Science Research Package questionnaire: doping susceptibility (use), legitimacy perceptions (perceived seriousness and effectiveness of the Saudi Sports Anti-Doping Authority in preventing PES use), morality and cheating measures, beliefs about the benefits of doping (perceived necessity for athletes to use PES to perform at the very highest levels), threat or deterrence appraisal (beliefs about the negative consequences of doping), beliefs about the reference group’s endorsement of doping (relevant others’ perceptions of them if they were caught using PES), authorities’ control of doping, and beliefs about other athletes’ attitudes toward doping.

The sample size was calculated as follows: given the size of the population (16,779), and allowing a margin of error of ±5% for determining the proportion of athletes with a positive predisposition to doping, the sample size required was 378.

### Data analysis

Data were analyzed using SPSS software, version 21.0 and AMOS statistical software. Descriptive statistics (mean, frequency, and percentage) were used to describe the quantitative and categorical variables. Internal consistency of latent variables was assessed using Cronbach’s alpha and convergent validity was evaluated using Pearson’s correlation coefficient for the items, subscale scores, and total scores. The construct validity latent variables was determined by using confirmatory factor analysis in which the correlation matrix, Kaiser-Meyer-Olkin (KMO) measurement of sampling adequacy, and Bartlett’s test of sphericity were used to assess the factorability of the items. Factor structure with five factors was used in the factor extraction process by using a principal component method. The proportion of variance explained by each of the factors was assessed through eigenvalues. Varimax rotation was used to obtain the rotated factors. The hypothesized model showing the relationship between the five variables and “performance-enhanced drug use” was quantified using structural equation modeling (SEM). Model fitness was assessed by using comparative fit index (CFI), incremental fit index (IFI), Tucker-Lewis index (TLI), the root mean square error of approximation (RMSEA), and the Bollen-Stine χ^2^-test. For RMSEA, values of ≤0.06 and ≤ 0.08 were used to consider the fitted model as excellent and acceptable, respectively. The 90% confidence intervals for RMSEA were used to assess the precision of point estimates. The values of ≥0.80 for CFI and IFI, and ≥ 0.85 for TLI were considered as having an acceptable and excellent fit to the data, respectively.

## Results

In total, 408 elite Saudi male football players aged between 16 and 40 years (*M* = 23.1) completed the survey (response rate = 68%). The baseline characteristics and demographics of the players are shown in Table 1. The majority of the participants had a secondary educational level and competed in football for more than five years (67.4 and 81.6%, respectively). A small proportion of the players (6.8%) participated in international football events, such as the Olympic Games or World Cup, with 64.2% belonging to Saudi national teams.

**Table 1:** Baseline characteristics and demographics of participants (*n* = 408)

	Frequency	%
Education level		
Below university	275	67.4
University and above	133	32.6
Years competing in football		
5 years or more	333	81.6
Less than 5 years	75	18.4
Highest level competed in football		
International	28	6.8
National	262	64.2
Local	118	29.0
Having therapeutic use exemption		
Yes	20	4.9%
No	388	95.1%
Have you ever been drug tested?		
Yes	118	28.9
No	290	71.1

With regard to doping susceptibility, 1.2% (*n* = 5) of the football players reported regularly using PES, 0.5% (*n* = 2) reported occasionally using PES for specific purposes, and 2.2% (*n* = 9) reported briefly using PES in the past. Thus, the prevalence of using PES in football players in this study is 3.9% (16 players). The total number of players who used PES or thought of using PES is 16 + 58 = 74, which equates to a prevalence rate of doping susceptibility of 17.1%. A small proportion (1.7%, *n* = 7) had the intention to use PES during the season.

With regard to legitimacy perceptions, the majority of the participants (70.3%, *n* = 287) believed that the Saudi Anti-Doping Committee treated all athletes equally, 72.5% (*n* = 296) believed that drug-testing procedures were secure, 67.4% (*n* = 275) were satisfied with a fair-hearing session for positive tests, and 24.5% (*n* = 100) of the sample thought that athletes who had been given therapeutic use exemptions had not been thoroughly evaluated and that their exemptions were not justified.

With regard to morality and cheating measures, 77.9% (*n* = 318) of the sample believed that deliberately using PES to improve performance was morally wrong under any circumstances. If a player was caught using PES or other methods, the majority (74.5%, *n* = 304) would feel ashamed, 80.0% (*n* = 328) would feel embarrassed, and 81.0% (*n* = 333) would feel very guilty.

With regard to beliefs about the benefits of doping, the majority of the players enjoyed the national celebrity status, lucrative financial sponsorship deals, personal best achievements, opportunities for remaining in the sport as a coach, trainer, or administrator, as well as future financial security and international celebrity status for performing well (82, 76, 84, 61, 83.9, 77.5%, respectively).

With regard to threat or deterrence appraisal*,* 65.0% (*n* = 265) believed that they were likely to be drug tested at least once a year. Less than half of the sample (44.0%, *n* = 180) thought that they were likely to get away with taking banned PES if they really tried. Of the participants, the majority (76.7%, *n* = 313) believed that the penalties for a positive drug test were severe.

Regarding beliefs about the reference group’s endorsement of doping when given the opportunity to use PES, 82% of players believed that coaches would disapprove, 85% that parents would disapprove, 66% that team mates would disapprove, and 84% that the team doctor would disapprove.

Relating to authorities’ control of doping, the majority of the players felt that the police and customs authorities were serious in preventing trafficking of banned PES (74 and 76%, respectively).

Concerning beliefs about other athletes’ attitudes toward doping, half (52.0%, *n* = 211) of the sample believed that less than 25% of the athletes in football sport engaged in doping to enhance their performance. In addition, the majority (71.8%, *n* = 293) of the participants believed that less than 25% of the coaches would encourage their athletes to use doping to enhance their performance (Table 2).

**Table 2:** Descriptive statistics of latent variables

Construct	Item(s)	Response scale (range)
Doping susceptibility	Which of the following most applies to you? (1) Never considered using a banned PES (2) At one stage thought briefly about using a banned PES (3) At one stage thought quite a bit about using a banned PES (4) Still think occasionally about using a banned PES because other athletes are using them (5) Briefly used a banned PES in the past but no longer do so (6) Occasionally use a banned PES now for specific purposes (7) Regularly try or use banned PES	1 = never to 7 = regular Doping susceptibility = 17.1% 74 participants answered yes to items 4 to 7
Legitimacy perceptions	How fair is the Saudi Anti-Doping Committee in terms of treating all athletes equally?	1 = Very fair to 5 = I do not know (287 participants answered fair = 70%)
	How secure is the Saudi Anti-Doping Committee’s drug-testing procedures? That is, in taking samples and the care of samples?	1 = Very secured to 5 = I do not know (296 participants answered secure = 72.5%)
	How satisfied are you that athletes in your sport who test positive will be given a fair hearing before a decision is made about applying a penalty?	1 = Very satisfied to 5 = I do not know (275 of the participants are satisfied = 67.5%)
	To what extent do you think that athletes who have been given Therapeutic Use Exemptions have been thoroughly evaluated and that their exemptions are justified?	1 = not justified to 5 = I do not know (100 participants answered not justified = 24.5%)
Morality and cheating measures	Which of the following statements best describes your own personal feelings about deliberately using banned PES? 1- I believe deliberately using banned PES to improve performance is morally wrong under any circumstances 2- I believe deliberately using banned PES to improve performance is morally OK under some circumstances, but wrong under others 3- I believe deliberately using banned PES to improve performance is morally OK under any circumstances	1 = *318* (77.9%) 2 = 75 (18.4%) 3 = 15 (3.6%)
	4- If you were caught, using banned performance-enhancing substances or methods, to what extent would you experience the following feelings? Shame/Embarrassment/Guilt.	1 = Not at all to 5 = great extent 304 (74.5%) would feel ashamed, 328 (80%) would feel embarrassed, 333 (81%) would feel guilty to a great extent.
Beliefs about the benefits of doping	How much would you personally like these outcomes for performing well in your sport? 1- National celebrity status 2- Lucrative financial sponsorship deals 3- Personal best achievements 4- Opportunities for remaining in the sport as coach, trainer, or administrator 5- Future financial security 6- International celebrity status	For each statement: 1 = a lot to 3 = not at all 1 = 342 (82%) like it a lot 2 = 308 (76%) like it a lot 3 = 343 (84%) like it a lot 4 = 24 9 (61%) like it a lot 5 = 338 (83.9%) like it a lot 6 = 316 (77.5%) like it a lot
*Threat or deterrence appraisal*	How likely it is that athletes at your level would be drug tested at least once a year?	1 = very likely to 5 = I do not know 265 (65%) answered likely
	If you were to take banned performance-enhancing substances how likely do you think that you could get away with it if you really tried to?	1 = very likely to 5 = I do not know 180 (44%) answered likely
	Are the penalties for a positive drug test in your sport severe or lenient?	1 = very severe to 5 = I do not know 313 (76.7%) answered severe
Beliefs about the reference group’s endorsement of doping	If you were given the opportunity to use a banned performance-enhancing substance, to what extent do you think each of the following people would approve or disapprove? Coach/parents/team mate//team doctor.	1 = Would definitely approve to 5 = Definitely disapprove 335 (82%) of participants think that the coach will disapprove 349 (85%) of parents would disapprove 269 (66%) of team mates would disapprove 346 (84%) of team doctors would disapprove
Authorities’ control of doping	How serious do you feel the following authorities are in preventing trafficking of banned performance-enhancing substances (police and customs)	1 = not at all to 5 = very serious 303 (74%) feel that police are serious 312 (76%) feel that customs are serious
Beliefs about other athletes’ attitudes toward doping	Out of 100%, how many athletes in football do you believe engage in doping to enhance their performance?	1 = 0–25% 2 = 26–50%, 3 = > 50% 1 = 211 (52%), 2 = 140 (34%), 3 = 57 (14%)

### Internal consistency

The internal consistency reliability of 25 items, with a combination of five latent variables, was assessed by calculating Cronbach’s α. The average measure of Cronbach’s α value of all 25 items is 0.77 and the Cronbach α values for the five latent variables ranges between 0.71 and 0.88. The values of the Cronbach alpha are close to the acceptable level of 0.70, which suggests a satisfactory estimate of the questionnaire’s reliability in this study (Table 3).

**Table 3:** Reliability (internal consistency) of 25 questionnaire items and five latent variables

Factors	No of items	Cronbach’s alpha	95% confidence interval
Legitimacy perceptions (5-point scale)	4	0.75	(0.70, 0.78)
Morality and cheating measures (5-point scale)	4	0.71	(0.68, 0.73)
Beliefs about the benefit of doping (3-point scale)	6	0.79	(0.76, 0.82)
Threat or deterrence appraisal (5-point scale)	3	0.74	(0.70, 0.77)
Beliefs about the reference group’s endorsement of doping (5-point scale)	8	0.88	(0.87, 0.90)
All items	25	0.77	(0.74, 0.80)

### Model fit

The chi-square minimum (CMIN) value of this model (71.647 (df = 10, *p* < 0.0001)) testing the difference between the observed data from the participants and hypothesized model, is highly statistically significant. The root mean square residual (RMR), which is an index of the amount of the estimated (by our model) variances and covariance, differs from the observed variances and co-variances and shows a value of 0.083, which is close to the acceptable level of < 0.10. The goodness of fit index (GFI), which identifies the proportion of the variance in the sample variance-covariance matrix, is accounted for by the model. Our model GFI and AGFI values at 0.948 and 0.891 are satisfactory as both values are close to the acceptable value (0.90) for a good model. The goodness of fit indices (comparative fit index, incremental fit index and Tucker-Lewis index) compare the default model to the independence model. All three indices of our model (CFI = 0.84, IFI = 0.79 and TLI = 0.86) are satisfactory. The root mean square error of approximation (RMSEA), which estimates lack of fit, is compared to the saturated model. Our model RMSEA value of 0.074 (90% CI = 0.065 to 0.0.89) indicates the acceptable fit of the model.

### Confirmatory factor analysis of four latent variables on a 5-point scale

The correlation among the 21 items of part of the instrument showed a statistically significant correlation. The KMO measures the sampling adequacy, which should be greater than 0.5 for a satisfactory factor analysis to proceed. The data show that the KMO measure of 0.822 and Bartlett’s tests of sphericity are significant (*p* < 0.0001). This indicates that the correlation matrix is not an identity matrix. The analysis of factor extraction with their eigenvalues, the percent of variance attributable to each factor, and the cumulative variance of the factors show that the first factor accounts for 42.1% of the variance, the second factor for 20.3%, the third factor for 14.1%, and the fourth factor for 10% with a cumulative variance of 86.5%. The loadings of the 21 items on the three factors were extracted. The higher the absolute value of the loading, the more the factor contributes to the variable. The loading indicates that four factors have contributed to each of the 21 items. The four factors are beliefs about the reference group’s endorsement of doping (eight items), legitimacy perceptions (four items), morality and cheating measures (four items), and threat or deterrence appraisal (three items).

**Fig. 1:**
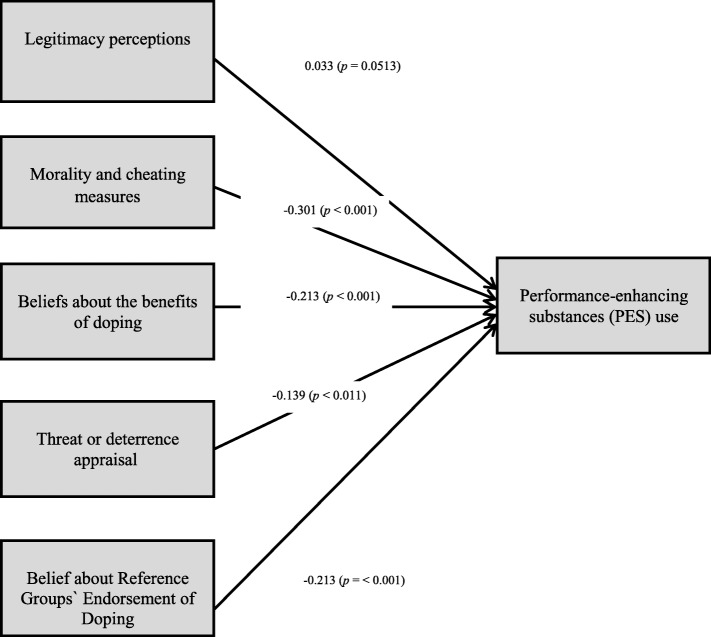
Structural equation model showing the relationship between the five variables and performance-enhancing substance use

Table 4 displays the parameter estimates of the variables using structural equation modeling: of the five variables, three variables (morality and cheating measures, threat or deterrence appraisal, and beliefs about the reference group’s endorsements) are related to performance-enhancing drug use. The estimates − 0.139, − 0.301, and − 0.213 indicate a negative relationship between these three variables and PES use. That is, for every one-unit increase in morality and cheating measures, the PES use decreases by − 0.139 units, and for every unit increase in threat or deterrence appraisal, the PES use decreases by − 0.301 units; for every one-unit increase in reference group endorsement, the PES use decreases by − 0.213 units. All three estimates are statistically significant (*p* < 0.011, *p* < 0.001 and p < 0.001) (Table 4). Figure 1 shows the structural equation model presenting the relationship between the five variables and PES use.

**Table 4:** Parameter estimates of the variables using structural equation modeling

Variables and their relation to performance-enhancing drug use	Estimate	S.E	Ratio (Estimate/SE)	*P*-value
Legitimacy perceptions	0.033	0.051	0.654	0.513
Morality and cheating measures	−0.139	0.054	−2.549	0.011
Beliefs about the benefits of doping	0.063	0.151	0.415	0.678
Threat or deterrence appraisal	−0.301	0.068	−4.426	< 0.001
Beliefs about the reference group’s endorsement	−0.213	0.058	−3.693	< 0.001

## Discussion

The overall prevalence rate of self-reported use of PES in elite Saudi football players is 3.9%, which is less than what is reported by Australian athletes [7]. The overall prevalence rate of doping susceptibility is 17.1%. Previously, we reported a prevalence rate of 4.3% in a countrywide survey in 1142 athletes from different types of sport (not limited to football players) in Saudi Arabia [2].

The majority of the players have a positive attitude toward the Saudi Anti-Doping Committee in terms of equal treatment of all athletes with acceptable drug-testing procedures and satisfactory hearing sessions. This positive attitude reflects the professional level of the committee as well as compliance with all the international standards and regulations of doping prevention. In comparison, Waddington et al. reported that 68% of English professional football players were aware and had a positive attitude of the national guidelines, with the remaining 32% not aware of the guidelines [8].

The majority of the players in the current study believe that using PES is morally wrong. They would feel shame and guilt if caught using PES because being caught would be detrimental to the reputation of the player and negatively affect his future. The majority of the players believe that they are likely to be drug tested at least once a year, that the penalties for a positive drug test are severe, and that they are not likely to get away with taking PES. The majority of the players believe that the people (such as coaches, team members, etc.) around them would disapprove of the use of PES.

When considering the five variables, PES use, or doping susceptibility is strongly correlated but negatively associated with morality and cheating measures, threat or deterrence appraisal, and beliefs about their reference group’s endorsement but not with legitimacy perceptions or beliefs about the benefits of doping. The strongest significant relationship was found between threat or deterrence appraisal and beliefs about their reference group’s endorsement regarding PES use. The strength of the association was significant but less strong for morality and cheating measures with PES use. The inverse relationship means that a player with higher morality and cheating measures and a higher threat or deterrence appraisal have less favorable attitudes toward using PES. The chance of PES use decreases by increases in the reference group endorsement.

In this study, there was a significant relationship between threat or deterrence appraisal and attitudes toward PES use. This means that if the player believes he can get away with cheating if tested, he is willing to cheat and has a more favorable attitude of doing so. This is consistent with the study of Gucciardi et al., which showed a significant but small relationship between threat appraisal and PES use [7].

The current study showed a strong association between beliefs about the reference group’s endorsement and PES use which means that the chance of PES use decreases by increases in reference group endorsement. In our knowledge, there are currently no other published studies related to beliefs about the reference group’s endorsement and PES use.

In the current study, we found an inverse relationship between morality and cheating measures and favorable attitudes toward using PES. Regarding morality and cheating measures, Donovan et al. were the first to develop a model which indicated that personal morality is a major input in an athlete’s attitudes and intentions to use PES [6]. Strelan et al. (2006) reported that the strongest predictor of PES use among athletes was moral beliefs and health concerns; but drug testing had no influence on the athlete’s decision [4]. Gucciardi et al. conducted a personality-profiling survey of 643 elite Australian athletes to determine their susceptibility to PES use. Morality, benefit appraisal, and threat appraisal had the strongest relationships with attitudes related to PES use. However, self-esteem, perceptions of legitimacy, and reference group opinion had non-significant associations with attitudes toward PES use. By using a mail survey of 1237 elite Australian athletes [7]. Jalleh et al. also reported that the significant predictors of attitudes toward PES use were morality, legitimacy, and reference group opinion while affordability and availability of the substances were not associated with actual PES use [9]. Lastly, in a cross-cultural investigation in three European countries (UK, Denmark and Greece), Kavussanu et al. studied the moral and achievement variables to predict the likelihood of doping in football players. The results indicated that higher moral values and a perceived low performance climate in a team of elite football players, the less likely the players are to use PES [10].

This study provides strong evidence for the importance of moral values and cheating measures in decisions related to PES use. As the relationship between PES use and morality is inverse in this study, we highly recommend the introduction of concepts of morality, and moral and ethical reasoning in anti-doping educational programs. Although the majority of the players in this study had a positive attitude related to the Saudi Anti-Doping Committee treating all athletes equally with secure drug-testing procedures and satisfactory hearing sessions, there was no significant relationship between legitimacy and attitude toward PES use. This is an unexpected finding, as it is generally believed that the stronger an organization’s perceived legitimacy, the more likely people will comply with that organization’s rules and regulations [11]. These findings are in contrast to the study of Donovan et al., reporting that if athletes perceive an anti-doping organization to be fair and its practices secure, it will enhance their compliance with anti-doping regulations and decrease PES use [6]. However, this seems not to be the case among Saudi football players. Our results are consistent with what reported by Kavussanu from the United Kingdom reporting that performance motivational climate and the moral atmosphere and identity were associated with an intention to dope. This means that athletes with a weaker moral identity and perceived a performance motivational climate, and a team environment with a tolerance to doping, were more likely to report the intention to use PES to enhance their performance and speed up recovery from injury [12].

A meta-analysis conducted by Ntoumanis et al. to determine the effect sizes of psychological and social-contextual factors on doping intentions, indicated that the use of food supplements, perceived social norms, and positive attitudes towards doping were the strongest positive correlates of doping intentions, while, morality and self-efficacy to refrain from doping had the strongest negative association with doping intentions and behaviors [13]. According to a systematic review conducted by Morente-Sanchez et al., there are limited research studies investigating the beliefs and knowledge about doping of football players, however, the authors reported the main reasons of athletes for doping are to improve performance, money gain, improving recovery and the idea that others are using PES [14]. Investigating 978 German elite athletes, Bloodworth et al. reported that the most prevalent reasons for PES use were to achieve athletic success (86%), followed by financial gain (74%). The acknowledged potential reasons for PES use were injury recovery and the economic pressures on an elite sportsperson [15].

In the current study, there was a no significant relationship between beliefs about the benefit of doping and attitude related to PES use indicating that achieving outcomes such as national or international celebrity status, financial gain, or opportunities for remaining in the sport as coach, trainer, or administrator are not the main predictors for Saudi players to use PES. Gucciardi et al. reported a contrasting finding as it was found that there was a significant relationship of moderate strength between attitudes toward PES use and appraisal of benefits [7].

The inconsistencies between the current study and other studies may be attributable to variations in the measurement of the five variables. However, they could be related to differences in culture, social background, level of professionalism, and differences in nationality between Saudi and Western athletes. The current study was limited to male football players compared with other studies including both genders and other types of sport. There is evidence to support the notion that athletes in different sports have a different approach to doping. Participants who regarded doping as a minor health risk seemed to be more often associated with doping compared to athletes who regarded doping as a significant health risk. Doping susceptibility is highest in speed and power sports, and lowest in sports requiring strong motor skills [16].

This study has several limitations, which should be considered when interpreting the results. It used a self-reporting format and due to the cross-sectional design, a causal relationship between PES use and the model variables could not be established. Although the response rate is 68%, this rate is acceptable in this type of survey. The WADA Social Science Research Package suggests that if the target population is elite athletes, acceptable response rates are 50% for face-to-face surveys, 40% for telephone surveys, and 25% for mail and online surveys [17]. It should be noted that the response rate in the Australian study was only 26%, however it was a mail survey [9]. It may be that PES use is higher among non-responding players; however, we believe that non-responses are because of lack of interest, being busy, or a lengthy questionnaire that are time consuming for the players. Finally, the study was limited to football and the male gender only.

## Conclusion

Although many educational and awareness programs have been developed and implemented to prevent doping in sport, these programs are not focused on the psychological behaviors, attitudes, and perceptions of athletes related to doping. The results of this study add new knowledge and highlights that there is a necessity to shift direction in the fight against doping from education and awareness programs to more in-depth analyses of factors and predictors underpinning PES use, and in particular the value of threats and morality as key factors in doping susceptibility.

## Data Availability

data available from the author upon request.

## Abbreviations

CFI: Comparative fit index
CMIN: Chi-square minimum
GFI: Goodness of fit index
IFI: Incremental fit index
IRB: Ethical Research Committee
KMO: Kaiser-Meyer-Olkin
PES: Performance enhancing substances
RMR: Root mean square residual
RMSEA: Root mean square error of approximation
SDCM: Sport Drug Control Model
SEM: Structural equation modeling
TLI: Tucker-Lewis index (TLI)
WADA: World Anti-doping Agency
